# Activity and Safety of NAB-FOLFIRI and NAB-FOLFOX as First-Line Treatment for metastatic Pancreatic Cancer (NabucCO Study)

**DOI:** 10.3390/curroncol28030164

**Published:** 2021-05-08

**Authors:** Elisa Giommoni, Evaristo Maiello, Vanja Vaccaro, Ermanno Rondini, Caterina Vivaldi, Giampaolo Tortora, Laura Toppo, Guido Giordano, Tiziana Pia Latiano, Cinzia Lamperini, Serena Pillozzi, Luca Boni, Lorenzo Antonuzzo, Francesco Di Costanzo

**Affiliations:** 1 Medical Oncology Unit, Careggi University Hospital, 50134 Florence, Italy; cinzia.lamperini@gmail.com (C.L.); serena.pillozzi@unifi.it (S.P.); lorenzo.antonuzzo@unifi.it (L.A.); dicostanzofrancesco@icloud.com (F.D.C.); 2 Medical Oncology Unit, IRCCS Casa Sollievo della Sofferenza, 71013 San Giovanni Rotondo, Italy; e.maiello@libero.it (E.M.); latiano.tiziana@virgilio.it (T.P.L.); 3 Medical Oncology Unit, Istituto Nazionale Tumori Regina Elena, 00144 Roma, Italy; vanja.vaccaro@hotmail.it; 4 Oncology Unit, Ospedale Santa Maria Nuova—IRCCS, 42100 Reggio Emilia, Italy; ermanno.rondini@gmail.com; 5 Medical Oncology Unit 2, Azienda Ospedaliero Universitaria Pisana, 56126 Pisa, Italy; caterina.vivaldi@gmail.com; 6 Medical Oncology Unit, Azienda Ospedaliera Universitaria Integrata, 37134 Verona, Italy; giampaolo.tortora@policlinicogemelli.it; 7 Medical Oncology Unit, ASST Cremona, 26100 Cremona, Italy; lauratoppomed@gmail.com; 8 Oncology Unit, Ospedale “Sacro Cuore di Gesù” Fatebenefratelli, 82100 Benevento, Italy; giordano.guido81@gmail.com; 9 Clinical Trial Coordinating Center, Careggi University Hospital, 50134 Florence, Italy; luca.boni@hsanmartino.it

**Keywords:** metastatic pancreatic cancer, nab-paclitaxel, FOLFIRINOX, dose finding

## Abstract

Background: Relevant improvement in first-line treatment of metastatic pancreatic cancer (mPC) was provided by FOLFIRINOX and by gemcitabine (gem) plus nab-paclitaxel (Nab-p) regimens. Regardless of the first-line treatment survival benefit, most patients survive less than 1 year. Aim: The objectives of this multicenter phase I/II study were to evaluate as first-line chemotherapy (CT) two modified regimens of FOLFIRINOX, replacing either oxaliplatin (Oxa) or irinotecan with Nab-p, in patients with mPC. Methods: The primary objectives of phase 1 were the definition of the dose limit binations, while for phase II they were the characterization of safety and activity of Nab-FOLFIRI and Nab-FOLFOX in mPC. Results: Sixty-three patients received Nab-FOLFIRI or Nab-FOLFOX in phase I. We defined MTD at 120 mg/m^2^ for Nab-p with FOLFIRI and 160 mg/m^2^ with FOLFOX. In phase II, we randomized 42 patients for each arm with the following results: (1) overall response rate (ORR) was 31% for both schedules; (2) a clinical benefit rate (CBR) of 69% and 71%; (3) 1-year survival was 41% and 50%; (4) progression free survival (PFS) was 6 months and 5.6 months; (5) median overall survival (OS) was 10.2 and 10.4 months for Nab-FOLFIRI and Nab-FOLFOX, respectively. (6) Neutropenia was the most common grade ≥3 adverse event in our regimens, significantly lower than that reported for the FOLFIRINOX triplet. Conclusion: Nab-FOLFIRI and Nab-FOLFOX might be hopeful first-line CT options for mPC patients, with promising activity and a good safety profile.

## 1. Introduction

PC is the seventh leading cause of cancer death worldwide in both sexes with about 331,000 deaths per year and an estimated 5-year survival rate of about 5%. Overall, about 53,670 new cases of PC were expected in the USA in 2017, while the annual incidence in the EU is 78,654 where it represents the fourth cause of death by cancer. Fifty percent of new diagnoses are in an advanced or M stage and more than 70% of the resected patients will experience a recurrence, with median OS ranging from 4 to 10 months [1,2,3,4].

Standard therapy for mPC is CT, which improves survival compared to the best supportive care according to a meta-analysis of seven randomized trials. For more than a decade, gem was the approved single agent treatment, while all other phase III trials failed to improve OS significantly [5,6,7].

Relevant improvement in the first-line treatment of mPC was provided by FOLFIRINOX (5-fluorouracil (5-FU) (antimetabolite drug), irinotecan (CPT11) (inhibitor of topoisomerase I), and oxaliplatin (Oxa) (exerts its cytotoxic effect through DNA damage)) and by gem (inhibitor of DNA synthesis) plus Nab-p (a microtubule stabilizing drug) regimens [8,9,10,11,12].

In the French trial (PRODIGE 4/ACCORD 11), the FOLFIRINOX combination obtained a better response rate (RR) and OS than gem alone. However, this combination showed significant toxicities with higher rates of grade ≥3 neutropenia with respect to gem alone [8].

Nab-paclitaxel (Nab-p), an albumin-bound formulation of paclitaxel [9], demonstrated a significantly longer OS in association with gem versus gem alone in the MPACT trial [10].

Regardless of the impact of first-line treatment on survival, most patients survive less than 1 year [3]. This poor prognosis highlights the need for new treatment options for mPC.

The Italian Cooperative Oncology Group for Clinical Research (GOIRC) designed a phase I/II trial to evaluate the replacement of either Oxa or CPT11 with Nab-p in the original FOLFIRINOX schedule, obtaining two new regimens: Nab-FOLFIRI (arm A) and Nab-FOLFOX (arm B). We postulated that these regimens would enhance activity with no additional toxicity compared to FOLFIRINOX.

The objectives of the phase Ib part were to define the dose limiting toxicities (DLTs) and maximum tolerated dose (MTD). In the second part, both schedules were evaluated in a multicenter randomized 1:1 open label phase II trial. The principal end points of phase II were to assess activity and safety of Nab-FOLFIRI and Nab-FOLFOX in patients with mPC as first-line CT.

## 2. Materials and Methods

### 2.1. Patients and Study Design

From February 2014 to October 2015, patients were enrolled from 7 Italian centers for phase I, while phase II patients were enrolled in 8 Italian sites from November 2015 to January 2017.

Eligibility criteria included age ≥18 and ≤75; a histological or cytological diagnosis of mPC with measurable disease defined according to the Response Evaluation Criteria in Solid Tumor (RECIST version 1.1 guidelines) [13] and no previous treatment for metastatic disease; an Eastern Cooperative Oncology Group (ECOG) PS score of 0 or 1, absence of previous abdominal radiotherapy on target lesions, absence of heart failure or unstable angina, or infarction within 12 months previous to inclusion; adequate organ function, including hematologic (absolute neutrophil count (ANC) ≥1.5 × 10^9^/L, platelets ≥100 × 10^9^/L), hepatic (bilirubin ≤1.5 times upper limits of normal (ULN), and renal (creatinine within normal limits ≤1.5 times (ULN)) values. Patients could have endoscopic or radiologic stenting to treat biliary obstructions.

Patients were not eligible if they had endocrine or acinar pancreatic carcinoma, known dihydropyrimidine dehydrogenase (DPD) deficiency, central nervous system metastases, other concomitant cancer, or history of cancer outside of carcinoma in situ of the cervix, basal, or squamous cell cancer; current active infections, serious pre-existing medical conditions or serious concomitant systemic disorders, a history of chronic diarrhea or inflammatory disease of the colon or rectum, or sub-occlusion or occlusion not resolved under symptomatic treatment; females who were pregnant or lactating were also excluded; and all subjects deprived of liberty or under guardianship or patients unable to undergo medical tests for geographical, social, or psychological reasons. Prior adjuvant treatment was allowed with tumor recurrence occurred ≥6 months after the last treatment.

Written informed consent was obtained from all patients before every study procedure.

The study was conducted in accordance with Good Clinical Practice (GCP), all regulatory requirements, and the principles of the Declaration of Helsinki. This study was submitted to and approved by the Ethics Committee of the cancer centers involved and also registered in the clinical trial network provided by US NIH (Clinicaltrials.gov NCT02109341).

#### 2.1.1. Phase I

Phase I was a non-randomized, open label, two arm dose-finding study for Nab-p in combination with FOLFIRI (l-folinic acid 200 mg/m^2^ intravenous (iv); 5-FU bolus 400 mg/m^2^ iv and 48 h continuous infusion (ci) 2400 mg/m^2^ at days 1 and 2; CPT11 180 mg/m^2^ iv at day 1) and FOLFOX (l-folinic acid 200 mg/m^2^ iv; 5-FU bolus 400 mg/m^2^ iv and 48 h ci 2400 mg/m^2^ at days 1 and 2; oxa 85 mg/m^2^ iv at day 1) every 2 weeks. The design used was the classical 3 + 3 design 13, with a dose escalation of 10 mg/m^2^ for each subsequent cohort. DLTs were treatment-related toxicities during the first cycle according to the National Cancer Institute Common Terminology Criteria of Adverse Event (CTCAE) ver 3.0 (https://ctep.cancer.gov/protocoldevelopment/electronic_applications/docs/ctcaev3.pdf, accessed on 4 September 2013). Dose escalation was stopped when one or more patients of a cohort of three patients experienced a DLT, and the dose below was declared MTD by a confirmatory cohort of three more patients.

#### 2.1.2. Phase II

In phase II, patients were randomized to receive Nab-p at a dose defined in phase I in association with FOLFIRI (arm A) (l-folinic acid 200 mg/m^2^ intravenous (iv); 5-FU bolus 400 mg/m^2^ iv and 48 h continuous infusion (ci) 2400 mg/m^2^ at days 1 and 2; CPT11 180 mg/m^2^ iv at day 1) and with FOLFOX (arm B) (l-folinic acid 200 mg/m^2^ iv; 5-FU bolus 400 mg/m^2^ iv and 48 h ci 2400 mg/m^2^ at days 1 and 2; oxa 85 mg/m^2^ iv at day 1) every 2 weeks. Patients continued treatment until disease progression or unacceptable toxicity, up to a maximum of 12 cycles.

The modified intention to treat the ITT population consisted of all patients who were randomized and received at least one dose of any component of study treatment. Patients were grouped according to the randomized treatment assignment. Patients treated during phase I were not included in this population. The safety population consisted of all patients who were randomized and received at least one dose of any component of study treatment. The activity was the primary outcome of interest. The response assessment was by computed tomography scan or magnetic resonance imaging (MRI) at baseline and every 8 weeks according to RECIST version 1.0. Clinical benefit rate (CBR) was estimated by dividing the total number of complete responses (CR), partial responses (PR), or stable disease (SD) by the number of patients included into the modified ITT population, and the exact 95% confidence intervals for each treatment arm were determined. PFS and OS were evaluated using Kaplan–Meier estimates of the median survival times (including 95% confidence intervals (CI)) and 6 month survival to inform assumptions for use in future trial planning.

#### 2.1.3. Study Assessment

Each patient enrolled in the study was evaluated at baseline with a physical exam, determination of ECOG PS, and clinical laboratory tests (blood count, sodium, potassium, alkaline phosphatase, alanine aminotransferase, aspartate aminotransferase, blood urea nitrogen, creatinine, glucose, albumin, lactate dehydrogenase, gamma glutamyltransferase, bilirubin). Tumor assessment at baseline was performed using a computed tomography scan or MRI as defined by RECIST.

Response was assessed every 8 weeks during treatment, then every three months during follow-up until progression of disease (PD), consent withdrawal, or death. Assessments included the following parameters: physical examination, clinical symptoms, laboratory or radiological findings, and tumor assessment by CT or MRI. All patients who received at least one dose of a study drug were evaluated for activity and safety. Safety was assessed by the incidence of treatment-related adverse events (AEs) according to CTCAE version 3.0.

Quality of life (QOL) was assessed using the European Organization for Research and Treatment of Cancer Quality of Life Questionnaire C30 (EORTC QLQ-C30, version 3.0). The EORTC QLQ-C30 is a 30 item questionnaire including five functional scales, three symptom scales, one QOL scale, and six single items on common symptoms. The EORTC QLQ-C30 questionnaire was distributed to the patients at baseline, then every two cycles and at the end of treatment. Questionnaires were scored according to the EORTC instructions.

## 3. Results

**Figure 1:**
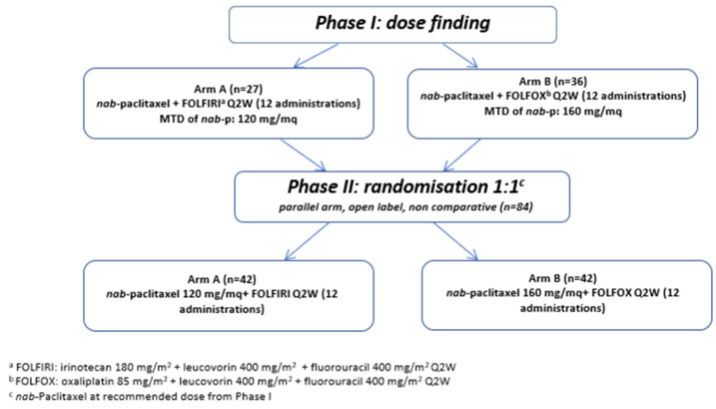
Flow chart of NabucCO study design.

NabucCO, a two-part multicenter phase I/II study, assessed as first-line CT two modified regimens of FOLFIRINOX, replacing either oxaliplatin or irinotecan with Nab-p (named Nab-FOLFIRI and Nab-FOLFOX arms), in patients with mPC (Figure 1).

We sought to develop a regimen that could potentially be better tolerated than FOLFIRINOX in mPC and, hence, could be administered for multiple cycles without the development of severe toxicity. For dose finding (phase I), the primary objectives were the definition of the DLTs and of the MTD of Nab-p in both combinations. For phase II, the primary objectives were further characterization of the safety and, in addition, the evaluation of the activity of Nab-FOLFIRI and Nab-FOLFOX in mPC in terms of ORR.

### 3.1. Phase I

From February 2014 to October 2015, 63 patients were enrolled in 8 Italian centers (on behalf of GOIRC) to receive either Nab-FOLFIRI (*n* = 27) or Nab-FOLFOX (*n* = 36) (Table 1). In the Nab-FOLFIRI arm the median age was 62 years (range 38–75) and 60 years (range 43–74) in the Nab-FOLFOX arm. DLTs during first cycle at the corresponding dose level are listed in Table 2. The MTDs for Nab-p were 120 mg/m^2^ in association with FOLFIRI and 160 mg/m^2^ with FOLFOX. The most common adverse events (AEs) of any grade, in the Nab-FOLFIRI and Nab-FOLFOX arms, respectively, were the following: neutropenia (52% and 81%), anemia (49% and 45%), thrombocytopenia (11% and 45%), nausea (56% and 47%), asthenia (56% and 53%), diarrhea (70% and 22%), liver toxicity (18% and 44%), and peripheral neuropathy (0% and 47%). In particular, the AEs ≥ grade 3 were neutropenia (45% and 31%), febrile neutropenia (4% and 11%), anemia (19% and 0%), liver toxicity (11% and 6%), and asthenia (7% and 8%). Two patients experienced a peripheral neuropathy of grade 3 (in the Nab-FOLFOX arm), a grade 3 liver function alteration was observed in 3 patients (2 with Nab-FOLFIRI treatment and 1 with Nab-FOLFOX), and a grade 4 toxicity was registered in 2 patients (1 patient for each schedule). The ORR were 18.5% (5/27) and 25% (9/36), respectively, in the two arms.

### 3.2. Phase II

In phase II (multicenter, randomized 1:1, open label), a total of 84 patients were recruited from November 2015 to January 2017 in 8 centers as reported above. Demographic and baseline disease characteristics of the patients are reported in Table 3. The median age was 60 years in the Nab-FOLFIRI arm (range 29–75) and 64 years in the Nab-FOLFOX arm (range 47–74). Most of the mPC patients enrolled in the Nab-FOLFIRI and Nab-FOLFOX arms had liver metastases (79% and 71%), a tumor of body/tail of pancreas (57% and 55%), ECOG PS was 0 for 25 patients (60%) and 29 patients (69%), respectively and score 1 for 17 (40%) and 13 (31%) patients. There were fewer patients with previous surgery (10% vs. 29%) and with previous adjuvant therapy (2% vs. 19%) in the Nab-FOLFOX arm.

Among 84 patients, 35 (42%) completed the planned treatment, 22 in the Nab-FOLFIRI arm and 13 in Nab-FOLFOX. Reasons for study discontinuation were disease progression (38%), clinical decision (6%), toxicity/adverse events (19%), patient’s refusal (2%), or death (1%).

**Figure 2:**
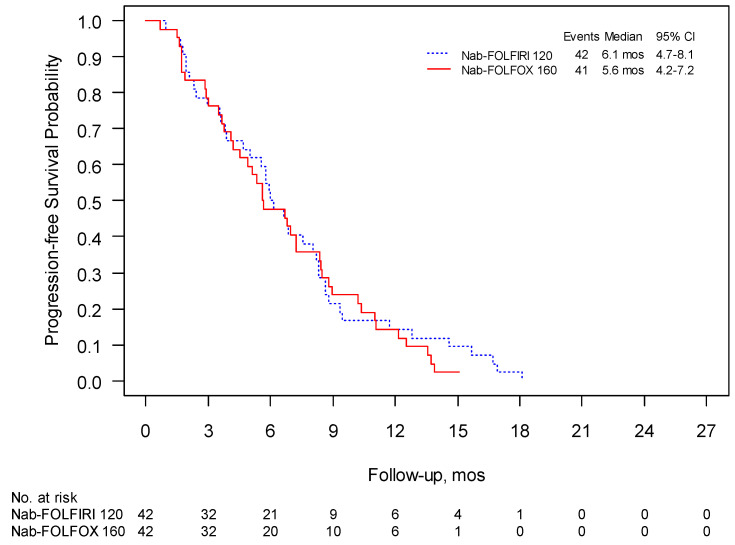
Phase II: progression free survival (PFS) was evaluated using Kaplan–Meier estimates of the median survival times (including 95% confidence intervals (CI)).

**Figure 3:**
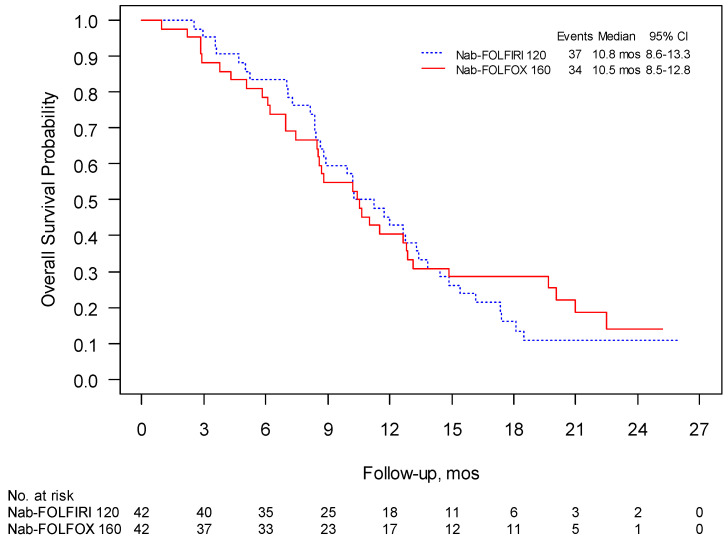
Phase II: overall survival (OS) was evaluated using Kaplan–Meier estimates of the median survival times (including 95% confidence intervals (CI)).

The 1-year survival was 41% for Nab-FOLFIRI and 50% for Nab-FOLFOX and the median follow-up was 15.4 and 18.8 months, respectively. PFS in these two regimens were 6.0 months (95% CI 4.7–8.1) and 5.6 months (95% CI 4.2–7.2), respectively, (Figure 2), while mOS was 10.2 (95% CI 8.6–13.3) and 10.4 months (95% CI 8.4–12.9) (Figure 3). The overall response rate (ORR) was 31% for both schedules with a clinical benefit rate (CBR) of 69% and 71%, respectively (Table 4).

The most common toxicities of any grade were anemia (83% and 55%), neutropenia (53% and 65%), nausea (38%), diarrhea (50% and 45%), mucositis (38% and 31%), fatigue (55% and 52%), and peripheral toxicities (36% and 71%). Grade 3–4 neutropenia occurred in 19% and 29%, with 12% and 10% of febrile neutropenia, respectively (Table 5). Grade ≥3 toxicities in Nab-FOLFIRI were neutropenia (19%) and febrile neutropenia (12%). In Nab-FOLFOX, main grade ≥3 toxicities were neutropenia (29%), fatigue (14%), and peripheral neuropathy (7%).

### 3.3. QoL

The EORTC QLQ-C30 questionnaire was used. Only 35% and 38% of patients in Nab-FOLFIRI and Nab-FOLFOX, respectively, completed all of the questionnaires planned at the 6th administration, and 16% and 21% completed all questionnaires during all of the treatments. Therefore, the analyses are only exploratory. Between the two arms, a minor deterioration of global health status was observed in arm A at the 6th cycle (61 baseline vs. 67.8 at 6th course), similarly to arm B (69.7 at baseline vs. 59.4 at 6th administration). Improvement in physical and emotional functioning was observed in arm A, along with a decrease in pain, fatigue, nausea, insomnia, anorexia, and constipation. An increase in diarrhea and fatigue was observed in arm B during the first 3 months of CT. The time until 5% definitive deterioration was not reached in either arms.

## 4. Discussion

PC incidence rates are quickly increasing in developed countries, with half of the patients being metastatic at diagnosis [2]. mPC is one of the most aggressive and highly lethal cancers, with a median life expectancy of around 1 year with current treatments, and the 5-year OS rate does not exceed 2% in Europe and the United States [4,5]. Current treatment options for mPC patients include monotherapy or combined therapy. FOLFIRINOX and gem plus Nab-p are both valid first-line options in the metastatic setting of PC [11], and recently, a meta-analysis comparing these two regimens did not provide evidence of a major benefit of one schedule over the other in terms of overall risk of death and progression [12]. A FOLFIRINOX regimen is recommended by ASCO and ESMO guidelines for mPC patients with either 0 or 1 ECOG PS, a favorable comorbidity profile, with good bilirubin values, and a good support system to care for the treatment-related additional toxicity [14]. Nonetheless, it has been primarily tested in relatively young patients and with good performance status. Even in this selected group, high rates of toxicity have been described.

As a consequence, modifications of this regimen have been tested to improve the toxicity profile of FOLFIRINOX. Recently, several studies on a small series of patients have been developed worldwide, testing modified regimens of FOLFIRINOX protocol (so-called modified FOLFIRINOX) in order to reduce toxicity [15] by suppression of the bolus 5-FU and decreasing dose of irinotecan or oxaliplatin [16,17,18,19,20]. Although many retrospective and thorough prospective pilot trials have been reported, no randomized trials evaluating modified FOLFIRINOX have been reported [15].

We attempted to develop a regimen triplet that could potentially be better tolerated than FOLFIRINOX in mPC and hence could be administered as first-line CT for multiple cycles without the development of severe toxicity. In the NabucCO study, FOLFIRINOX was revised by replacing irinotecan or oxaliplatin with Nab-p to obtain two novel schedules (Nab-FOLFIRI and Nab-FOLFOX) that could be as effective and less toxic than the triplet used in the PRODIGE/ACCORD11 study. A rationale to combine platinum, fluoropyrimidine, and a taxane emerges from studies in gastric and head/neck cancer evidencing increased response and survival [21]. In addition, Safran et al. described a phase I Nab-FOLFOX in advanced PC (both locally advanced and metastatic patients) (MTD Nab-p 150mg/m^2^), and a phase II trial is still ongoing [22]. Nab-p is a target CT with a more selective action that uses nanotechnology and shows a good efficacy and safety profile in mPC [23,24]. In phase I, we determined MTD and DLTs of Nab-p to demonstrate that Nab-FOLFIRI and Nab-FOLFOX are feasible and reasonably tolerated. In phase II, we achieved an ORR of 31% for both regimens with a CBR of 69% and 71% for Nab-FOLFIRI and Nab-FOLFOX, respectively. Despite the limitations of a comparison between different trials, we can state that the results in terms of RR are similar to data reported by Conroy and colleagues [8] and better than the meta-analysis of retrospective and phase II studies by Thibodeau et al. [25] that reported an overall pooled RR of 24.5% (95% CI: 16.92–32.15%). The median PFS (6 and 5.6 months) and OS (10.2 and 10.4 months) in our trial are similar to those reported in the PRODIGE4/ACCORD11 trial [8,24].

The NabucCO trial was designed with eligibility criteria quite similar to the Conroy study [8] in order to enroll comparable populations. The median age of patients in the NabucCO study was 60 in the Nab-FOLFIRI arm, 64 in the Nab-FOLFOX arm, and 61 in the FOLFIRINOX trial. The site of the primary tumor was mainly the head of the pancreas in NabucCO phase II with 43% (18/42) and 45% (19/42), 17% (7/42) and 14% (6/42) of biliary stenting, and 79% (33/42) and 71% (30/42) of patients with hepatic metastasis for Nab-FOLFIRI and Nab-FOLFOX, respectively. In the PRODIGE4/ACCORD11 trial, 39.2% of patients had a primary tumor in the head of the pancreas, 14% of them had a biliary stenting at the time of enrollment, and 87% had hepatic metastasis. Overall, these data suggest that the NabucCO population had an important percentage of unfavorable factors that could have worsened the safety of treatment in line with the PRODIGE4/ACCORD11 population.

In the NabucCO phase II trial, the median cycles of treatment were 9 (range 1 to 12) and 8 (range 1 to 12) for Nab-FOLFIRI and Nab-FOLFOX, respectively, suggesting a good compliance to therapy. In the PRODIGE4/ACCORD11 trial, the median of cycles was 10, while Abrams et al. reported a median duration of chemotherapy with FOLFIRINOX of 100 days (7 cycles) in a U.S.-based cohort of 609 patients with mPC [25].

With regard to hematologic G3/4 toxicity, neutropenia is the most common AE in our regimens, with 19.1 and 28.6 in Nab-FOLFIRI and Nab-FOLFOX, respectively; this rate is significantly lower than that reported in the FOLFIRINOX trial (45.7% G3/4 neutropenia) [8].

In the Conroy trial, the occurrence of severe diarrhea (12.7%) and vomiting (14.5%) during the first cycles of treatment had a negative impact on the overall quality of life of mPC patients [8]. Notably, among non-hematologic toxicity in both of our studies, diarrhea was strongly reduced (2.4% and 0.0%) and vomiting was not registered; again, our schedules compare favorably with FOLFIRINOX in terms of gastrointestinal AE.

Nab-p nowadays is not registered in Europe for use over first-line of treatment, and triplet is used only in a subgroup of patients with good PS and rarely in successive settings due to the rapid deterioration of clinical conditions of patients after first-line treatment. Therefore, NabucCO regimens could represent potential treatments in order to give a possibility to use either triplets and Nab-p as first-line CT. On the whole, both the activity and tolerability of both proposed triplet regimens is promising, with a low rate and severity of AEs. Therefore, Nab-FOLFIRI and Nab-FOLFOX might be promising first-line CT options for mPC patients, though further studies are required to explore the potential of these novel triplets in phase III settings.

## Figures and Tables

**Table 1:** Phase I: patient’s characteristics.

Patient’s Characteristics	Nab-FOLFIRI (Arm A)		Nab-FOLFOX (Arm B)	
	*N*	%	*N*	%
**Sex**				
Male	15	56	14	39
Female				
**Age, years**				
Median	62		60	
Range	(38–75)		(42–74)	
**ECOG**				
0	19	70	23	64
1	8	30	13	36
**Prior adjuvant therapy**				
Yes	2	7	4	11
No	25	93	32	89
**Prior surgery on primary**				
Yes	7	26	9	25
No	20	74	27	75
**Tumor localization**				
Head	14	52	15	42
Neck–body	13	48	21	58
**No. of metastatic sites**				
1	6	22	17	47
2	12	44	15	42
≥3	9	34	4	11
**Site of metastatic disease**				
Liver	20	74	28	78
Lung	8	30	9	25
Lymph nodes	18	49	16	45
Peritoneum	6	22	8	22
Other	5	19	0	0
**Biliary stenting**				
Yes	5	19	4	11
No	22	81	32	89
**Ca 19.9 baseline**				
Median	68.3		336.5	
Range	(0.95–41,251)		(0.80–136,505)	

**Table 2:** Phase I: dose escalation and DLTs.

Nab-p (mg/m^2^)	Level	No. Pts	Nab-FOLFIRI (Arm A)	No. pts	Nab-FOLFOX (Arm B)
90	I	3		3	
100	II	3		3	
110	III	6	Liver toxicity G3 (1pt)	3	
120	IV	6	MTD	3	
130	V	3 + 3 ↑↓	Neutropenia G4, leucopenia G3 (1 pt) anemia G3, neutropenia G4, thrombocytopenia G3, and mucositis G2 (1 pt)	3	
140	VI	3	Neutropenia G4, leucopenia G3, and thrombocytopenia G3 (1 pt) Fever G1, asthenia G3, and hospitalization (1 pt) DLT	6	Mucositis G3, diarrhea G3, and anorexia G3 (1 pt)
150	VII	NA		6	
160	VIII	NA		6	Nausea G3 (1 pt) *MTD*
170	*IX*	NA *		3	Sepsis (1 pt) Febrile neutropenia G4 and leucopenia G3 (1 pt) DLT

* NA = not applicable.

**Table 3:** Phase II: patient’s characteristics.

Patient’s Characteristics	Arm A (Nab-FOLFIRI)		Arm B (Nab-FOLFOX)	
	*N*	%	*N*	%
**Sex**				
Male	26	62	27	64
Female	16	38	15	36
**Age**				
Median	60 (range 29–75)		64 (range 47–74)	
**ECOG**				
PS 0	25	60	29	69
PS 1	17	40	13	31
**Site of primary**				
Head	18	43	19	45
Body/tail	24	57	23	55
**Previous surgery**				
Yes	12	29	4	10
**Previous adjuvant treatment**				
Yes	8	19	1	2
**Biliary stenting**	7	17	6	14
M + liver	33	79	30	71
**Ca 19.9 (U/mL)**				
median	566 (range 3–634,657)		1101 (range 0–167,350)	

**Table 4:** Phase II: best overall response and clinical benefit rate.

BEST OVERALL RESPONSE	ARM A (Nab-FOLFIRI)		ARM B (Nab-FOLFOX)	
	*N*	%	*N*	%
CR	0	0	0	0
PR	13	31	13	31
SD	16	38	17	40
**PD**	11	26	7	17
Not assessed	2	5	5	12
**CLINICAL BENEFIT RATE**				
CR + PR + SD	29	69	30	71
Other	13	31	12	29

**Table 5:** Most relevant grade 3 or 4 adverse events and historical controls. NR: not reported.

Phase II: Most Relevant Grade 3 or 4 Adverse Events and Historical Controls.						
Event	Nab-FOLFIRI *n*	Nab-FOLFIRI %	Nab-FOLFOX *n*	Nab-FOLFOX %	FOLFIRINOX Conroy, 2011 *n* (%)	Nab-FOLFOX Safran, 2015 *n* (%)
Hematologic						
Neutropenia	8	19.1	12	28.6	75/164 (45.7)	6/35 (17.1)
Febrile neutropenia	5	11.9	4	9.5	9/166 (5.4)	NR
Thrombocytopenia	1	2.4	0	0	15/166 (9.1)	0/35 (0.0)
Anemia	3	7.1	4	9.5	13/166 (7.8)	2/35 (5.7)
Non hematologic						
Fatigue	2	4.8	6	14.3	39/165 (23.6)	9/35(25.7)
Vomiting	0	0.0	0	0.0	24/166 (14.5)	0/35 (0.0)
Diarrhea	1	2.4	0	0	21/165 (12.7)	2/35 (5.7)
Sensory neuropathy	0	0	3	7.1	15/166 (9.0)	2/35 (5.7)
Liver toxicity	2	4.8	1	2.4	12/165 (7.3)	1/35 (2.9)
Thromboembolism	0	0.0	0	0.0	11/166 (6.6)	0/35 (0.0)

## Data Availability

Data available on request to the corresponding author.
